# Effective Phase‐Alignment for 2D Halide Perovskites Incorporating Symmetric Diammonium Ion for Photovoltaics

**DOI:** 10.1002/advs.202001433

**Published:** 2021-05-24

**Authors:** Yalan Zhang, Jialun Wen, Zhuo Xu, Dongle Liu, Tinghuan Yang, Tianqi Niu, Tao Luo, Jing Lu, Junjie Fang, Xiaoming Chang, Shengye Jin, Kui Zhao, Shengzhong (Frank) Liu

**Affiliations:** ^1^ Key Laboratory of Applied Surface and Colloid Chemistry Ministry of Education Shaanxi Key Laboratory for Advanced Energy Devices Shaanxi Engineering Lab for Advanced Energy Technology School of Materials Science and Engineering Shaanxi Normal University Xi'an 710119 China; ^2^ Dalian National Laboratory for Clean Energy iChEM Dalian Institute of Chemical Physics Chinese Academy of Sciences Dalian 116023 China; ^3^ University of the Chinese Academy of Sciences Beijing 100039 China

**Keywords:** charge transfer, diammonium ion, perovskite solar cells, phase alignment

## Abstract

New structural type of 2D AA′*_n_*_−1_M*_n_* X_3_ *_n_*_+1_ type halide perovskites stabilized by symmetric diammonium cations has attracted research attention recently due to the short interlayer distance and better charge‐transport for high‐performance solar cells (PSCs). However, the distribution control of quantum wells (QWs) and its influence on optoelectronic properties are largely underexplored. Here effective phase‐alignment is reported through dynamical control of film formation to improve charge transfer between quantum wells (QWs) for 2D perovskite (BDA)(MA)*_n_*_‐1_Pb*_n_* I_3_ *_n_*_+1_ (BDA = 1,4‐butanediamine, 〈*n*〉 = 4) film. The in situ optical spectra reveal a significantly prolonged crystallization window during the perovskite deposition via additive strategy. It is found that finer thickness gradient by *n* values in the direction orthogonal to the substrate leads to more efficient charge transport between QWs and suppressed charge recombination in the additive‐treated film. As a result, a power conversion efficiency of 14.4% is achieved, which is not only 21% higher than the control one without additive treatment, but also one of the high efficiencies of the low‐*n* (*n* ≤ 4) AA′*_n_*_−1_M*_n_* X_3_ *_n_*_+1_ PSCs. Furthermore, the bare device retains 92% of its initial PCE without any encapsulation after ambient exposure for 1200 h.

## Graphical abstract

The in situ optical spectra reveal a significantly prolonged crystallization window during the perovskite deposition via additive strategy. Finer thickness gradient by n values in the direction orthogonal to the substrate leads to more efficient charge transport between quantum wells and suppressed charge recombination in the additive‐treated film. Finally, the power conversion efficiency of 14.4% is obtained.

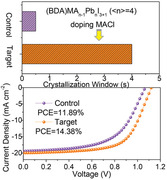

## 1 Introduction

Organic and inorganic halide perovskites as next‐generation photovoltaic materials have advantages such as high absorption coefficients,^[^ ^1^, ^2^, ^3^ ^]^ long carrier life,^[^ ^6^, ^7^ ^]^ large carrier mobility,^[^ ^7^, ^8^ ^]^ and simple fabrication methods.^[^ ^2^, ^9^, ^10^ ^]^ The photovoltaic efficiency achieved based on this material has exceeded 25%^[^ ^11^ ^]^ and is being scaled‐up gradually.^[^ ^12^, ^13^ ^]^ However, the long‐term stability of 3D perovskites is extremely unsatisfactory when exposed to humidity, heat, and light, which is a fatal drawback to their real‐world application.^[^ ^14^, ^15^ ^]^ Common methods to improve their stability include interface engineering, encapsulation, and exploration of new photoactive materials. In recent years, solar cells based on 2D organometal halide perovskites have shown promising long‐term stability,^[^ ^16^, ^17^, ^18^, ^19^, ^20^, ^21^, ^22^, ^23^ ^]^ and the efficiency has been continuously improved, but it still lags behind their 3D counterparts, partially due to the quantum confinement and low carrier transport of the quantum well (QW) structure.

Nowadays, much progress has been achieved to improve carrier transport for the (A)_2_A′*_n_*_‐1_M*_n_* X_3_ *_n_*_+1_ 2D perovskites and AA′*_n_* M*_n_* X_3_ *_n_*_+1_ 2D perovskites.^[^ ^18^, ^19^, ^24^, ^32^, ^33^, ^34^, ^35^, ^36^, ^37^, ^38^, ^39^, ^40^, ^41^, ^42^ ^]^ For example, the (BA)_2_MA_3_Pb_4_I_13_ (BA = *n*‐butylamine) 2D perovskite solar cell fabricated by Mohite et al. achieved a PCE of 12.52%,^[^ ^35^ ^]^ confirming that the out‐of‐plane QWs formed by hot‐casting are more beneficial to charge transfer. The (BA)_2_(MA)_3_Pb_4_I_13_ perovskite doped with Cs^+^ further improved the PCE to 13.7% and has superior humidity stability.^[^ ^18^ ^]^ Many papers have been published on the control of the orientation and thickness distribution of QWs, as well as their influences on charge transfer between QWs.^[^ ^21^, ^23^, ^33^, ^34^, ^38^, ^41^, ^42^, ^43^, ^44^, ^45^, ^46^, ^47^ ^]^ For example, the (BA)_2_(MA)_3_Pb_4_I_13_ thin film exhibits an inherent phase segregation wherein thicker QWs were mainly located close to the top surface and thinner QWs concentrated on the bottom.^[^ ^39^ ^]^ The well control of phase segregation has led to a record PCE of over 18%.^[^ ^34^ ^]^ Kanatzidis et al. first reported the AA′*_n_* M*_n_* X_3_ *_n_*_+1_ perovskite,^[^ ^31^ ^]^ after which we not only systematically delved into the complex crystallization kinetics during fast solution‐deposition,^[^ ^32^ ^]^ but also got the highest PCE of 18.5% via improving thickness distribution of QWs in the perpendicular direction and charge transfer between QWs.^[^ ^33^ ^]^ These excellent pioneer works highlight a gleaming but indispensable stage for the understanding and control of the QW thickness distribution that are crucial to charge transfer and the performance of the optoelectronic devices.

However, unlike (A)_2_A′*_n_*_‐1_M*_n_* X_3_ *_n_*_+1_ and AA′*_n_* M*_n_* X_3_ *_n_*_+1_ 2D perovskites, there are few reports on the thickness distribution and charge transfer of the AA′*_n_*_−1_M*_n_* X_3_ *_n_*_+1_ type 2D perovskites, where A′ is diammonium cations. The organic cations in 2D perovskites have been shown to keep water molecules away from the reaction site by straining the Pb—I bond on the surface.^[^ ^48^, ^49^, ^50^, ^51^, ^52^, ^53^ ^]^ In addition, the new structure incorporating diammonium ions is more conducive to charge transport because the distance between the inorganic layers is shorter and has more 3D‐like characteristics in contrast to the (A)_2_A′*_n_*_‐1_M*_n_* X_3_ *_n_*_+1_ 2D perovskites.^[^ ^20^, ^26^, ^27^, ^28^, ^29^, ^30^, ^54^ ^]^ Although molecular‐chemistry‐dependent structure and optoelectronic properties of the AA′*_n_*_−1_M*_n_* X_3_ *_n_*_+1_ type 2D perovskites have been demonstrated, unfortunately, a series of low‐*n* AA′*_n_*_−1_M*_n_* X_3_ *_n_*_+1_ perovskite devices reported currently shows poor performance. For example, solar cells achieved PCEs of 4.2%, 12.0%, 7.1%, 13.0%, 13.3%, 13.8%, 15.0%, 15.2% and 15.6% based on *n* ≤ 4 AA′*_n_*_−1_M*_n_* X_3_ *_n_*_+1_ perovskites incorporating 3‐(aminomethyl)piperidinium (4AMP^2+^),^[^ ^27^ ^]^ 4‐(aminomethyl)piperidinium (3AMP^2+^),^[^ ^46^ ^]^ 1,4‐phenylenedimethanammonium (PDMA),^[^ ^55^ ^]^ 1,3‐propanediamine (PDA),^[^ ^20^, ^56^, ^57^ ^]^ trans‐1,4‐cyclohexanediamine (CHDA),^[^ ^58^ ^]^ p‐xylylenediamine (PXD),^[^ ^59^ ^]^ and 3‐(dimethylammonium)‐1‐propylammonium (DMAPA^2+^).^[^ ^60^ ^]^ In contrast to the heavily reported (A)_2_A′*_n_*_‐1_M*_n_* X_3_ *_n_*_+1_ and AA′*_n_* M*_n_* X_3_ *_n_*_+1_ perovskites, the much worse performance of the AA′*_n_*_−1_M*_n_* X_3_ *_n_*_+1_ solar cells is caused by the lack of systematic investigations on how to control the formation and distribution of QWs. Therefore, it is particularly important to go beyond molecular chemistry to examine film formation and its influence on the QW thickness distribution, which is expected to play a crucial role in charge transfer and trap formation between QWs.

Herein, we demonstrate understanding of phase‐alignment via dynamical control of film formation and its influence on charge transfer kinetics and trap formation for the (BDA)(MA)*_n_*_‐1_Pb*_n_* I_3_ *_n_*_+1_ (*n* = 4) QWs. The in situ UV–vis examination during the perovskite deposition reveals almost the same solid film formation but much slower crystallization kinetics with addition of the methylammonium chloride (MACl) additive. The slow crystallization process results in the increase of grain size and crystallinity, and the film surface is smoother, which is important for the photoelectric performance of the film. The orientation of QWs in the MACl‐doped film is improved as indicated by XRD, making charge transfer more efficient in the direction orthogonal to the substrate. Besides, the finer distribution of QW thickness and the effective extraction form of carrier from low‐*n* QWs to high‐*n* QWs in the layered perovskite films were confirmed by transient absorption spectroscopy (TA), showing that the fastest transfer is achieved in the 6 mg mL^−1^ MACl‐doped film. Moreover, the carrier recombination is suppressed due to the reduction of defects. In the end, the PCE_max_ increases greatly from 11.89% to 14.38%, which stands one of the high values for the *n* ≤ 4 AA′*_n_*_−1_M*_n_* X_3_ *_n_*_+1_ PSCs. Furthermore, the bare device without any encapsulation retains 92% of its initial PCE after being exposed to ambient for 1200 h.

## 2 Results and Discussion

### 2.1 Photovoltaic Performance

**Figure 1:**
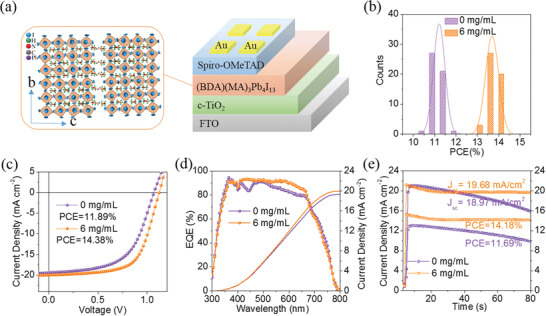
a) The solar cell architecture and crystal structure of the 2D perovskite (BDA)(MA)*_n_*_‐1_Pb*_n_* I_3_ *_n_*_+1_ (〈*n*〉 = 4). b) PCE histogram of the control and optimized (with 6 mg mL^−1^ MACl) devices determined from 50 cells of each type. c) *J–V* curves d) EQE and integrated *J*_sc_ for the control and optimized (with 6 mg mL^−1^ MACl) devices. e) Stabilized output power under maximum power point tracking (AM 1.5G, 100 mW cm^–2^) in ambient conditions over 80 s for the control and optimized (with 6 mg mL^−1^ MACl) devices.

The AA′*_n_*_−1_M*_n_* X_3_ *_n_*_+1_ type 2D halide perovskites (BDA)(MA)*_n_*_‐1_Pb*_n_* I_3_ *_n_*_+1_ (〈*n*〉 = 4) stabilized by symmetric diammonium cations BDA^2+^ were fabricated using a hot‐spinning method on a 100 °C substrate that has been commonly used to deposit (A)_2_A′*_n_*_‐1_M*_n_* X_3_ *_n_*_+1_ 2D perovskite films, the annealing condition is 125 °C for 10 min. Planar solar cells were fabricated with the architecture of FTO/c‐TiO_2_/perovskite/spiro‐OMeTAD/Au (**Figure** **1**a). The average PCE (PCE_ave_) values are 11.2 ± 0.27%, 12.6 ± 0.36%, 13.9 ± 0.22%, and 10.5 ± 0.43% with the MACl concentration increases from 0 to 3, 6, and 10 mg mL^−1^, respectively (**Table** **1**). Figure 1b shows the PCE statistic distribution, from which we can see that the efficiency of optimized device (with 6 mg mL^−1^ MACl) is significantly improved and the distribution is lumped, showing excellent repeatability. The optimized device (with 6 mg mL^−1^ MACl) obtains a best PCE (PCE_max_) of 14.38%, which is not only higher than the control case (11.89%), but also among the high efficiencies for the low‐*n* (*n* ≤ 4) AA′*_n_*_−1_M*_n_* X_3_ *_n_*_+1_ PSCs (Figure 1c). The significant enhancement in PCE is mainly due to the improvements in the *V*_oc_ (1.12 vs 1.06eV) and FF (57.4% vs 64.2%), while *J*_sc_ shows a negligible variation (with ≤6 mg mL^−1^). However, we observed deterioration in all parameters of the device (with 10 mg mL^−1^ MACl). External quantum efficiency (EQE) spectra of the devices are illustrated in Figure 1d, and the mismatch between the integrated current and *J*_sc_ is negligible. Besides, the EQE of the optimized device is higher and flatter in the range of 400–700 nm in contrast to the control one, which means more efficient charge collection. The stabilized power output measured at a fixed maximum power point (MPP) voltage under continuous AM 1.5‐G, 1‐sun illumination indicates that the optimized solar cells not only has higher output efficiency (14.18% vs 11.69%) and current (19.68 vs 18.97 mA cm^–2^), but also better illumination stability (Figure 1e). We also noticed in the experiment that the device performance is also closely related to the substrate temperature during solution‐casting (Table S1, Supporting Information), the annealing temperature (Figures S1 and S2 and Table S2, Supporting Information), the solvent property and solution concentration (Tables S3 and S4, Supporting Information).^[^ ^61^ ^]^

**Table 1:** Summaries of the photovoltaic parameters of devices based on the (BDA)(MA)*_n_*_‐1_Pb*_n_* I_3_ *_n_*_+1_ (〈*n*〉 = 4) with the additive concentrations of 0, 3, 6, and 10 mg mL^−1^

Amount [mg mL^−1^]		*V*_oc_ [V]	*J*_sc_ [mA cm^–2^]	FF [%]	PCE [%]
0	Average	1.05 ± 0.01	19.1 ± 0.29	55.9 ± 0.99	11.2 ± 0.27
	Max	1.06	19.4	57.4	11.89
3	Average	1.07 ± 0.01	19.8 ± 0.33	59.4 ± 0.93	12.6 ± 0.36
	Max	1.08	20.1	61.0	13.25
6	Average	1.11 ± 0.01	19.6 ± 0.19	63.5 ± 0.89	13.9 ± 0.22
	Max	1.12	19.9	64.2	14.38
10	Average	1.09 ± 0.02	16.8 ± 0.29	57.5 ± 1.70	10.5 ± 0.43
	Max	1.09	17.0	61.0	11.32

### 2.2 Crystallinity and Morphology

**Figure 2:**
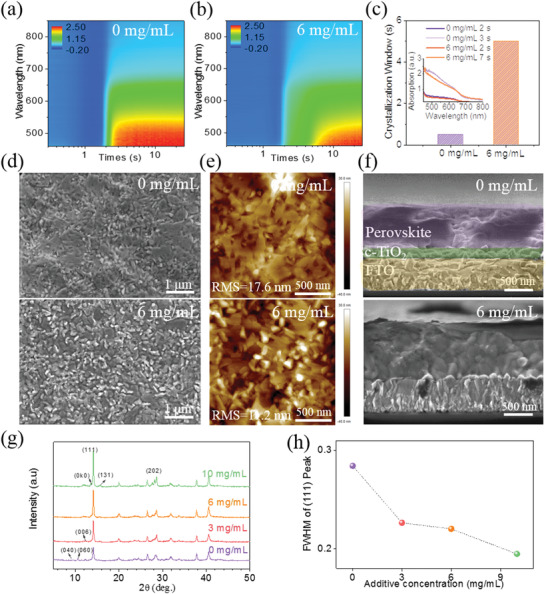
a,b) In situ UV–vis absorption spectra for the 2D (BDA)(MA)*_n_*_‐1_Pb*_n_* I_3_ *_n_*_+1_ (〈*n*〉 = 4) perovskite films during the deposition with and without MACl. c) Crystallization window of the 2D (BDA)(MA)*_n_*_‐1_Pb*_n_* I_3_ *_n_*_+1_ (〈*n*〉 = 4) films with and without MACl; the inset shows the absorption spectra at the onset and end of crystallization. d) Plan‐view scanning electronic microscopy (SEM) images, e) Atomic force microscope (AFM) images, and f) Cross‐sectional SEM images of the control and optimized (with 6 mg mL^−1^ MACl) films. g) X‐ray diffraction (XRD) patterns and h) The FWHM of the (111) diffraction peak of the films with various additive concentrations.

In an attempt to determine the source of the increased *V*_oc_ and FF, we first studied the effects of the MACl additive on formation of the (BDA)(MA)*_n_*_‐1_Pb*_n_* I_3_ *_n_*_+1_ (〈*n*〉 = 4) films. Due to fast evaporation of the working solvent on the hot substrate, we observed a fast solidification and crystallization during the perovskite deposition, which was evidenced by in situ UV–vis absorption analysis (**Figure** **2**a).^[^ ^62^ ^]^ The perovskite begins to crystallize from disordered sol–gel at the spinning time of ≈2 s, with a quick increase in the absorption intensity. The absorption intensity reaches a peak at ≈2.5 s, without any noticeable change afterwards. This indicates a crystallization window of only ≈0.5 s. However, from the point of view of material growth kinetics, such rapid crystallization, which is far from a thermodynamical process, generally leads to perovskite films with low crystallinity, high defect density, and a random distribution of QW thickness. To improve QW formation, we decouple the film solidification and perovskite crystallization processes by introducing a low concentration of methylammonium chloride (MACl) into the precursor solution. In situ optical observation indicates an almost identical spinning time for the onset of film solidification and perovskite crystallization of ≈2 s with the addition of 6 mg mL^−1^ MACl (Figure 2b). However, the MACl treatment leads to a much slower structural evolution in contrast to the control. The absorption intensity reaches a peak at a spinning time of ≈7 s, leading to a crystallization window of ≈5 s (Figure 2c) for the MACl‐treated case, which is significantly longer than that for the control (≈5 vs ≈0.5 s). This indicates that the additive gives more time for perovskite nucleation and crystallization during the deposition.

It is necessary to evaluate how the crystallization window influences the formation of QWs, so we carried out a series of analyses on the final films with MACl concentrations of 0, 3, 6, and 10 mg mL^−1^, respectively. The results of the plan view scanning electron microscope (SEM) show that the control has poor uniformity (Figure 2d, Figure S3a, Supporting Information), in which large grains of about 1–2 µm are interspersed within a film consisting of 200–300 nm grains, and the root‐mean‐square roughness (RMS) measured by atomic force microscope (AFM) is 17.6 nm (Figure 2e, Figure S3b, Supporting Information). This phenomenon was previously observed in 2D (GA)(MA)*_n_* Pb*_n_* I_3_ *_n_*_+1_ (〈*n*〉 = 3) perovskite films,^[^ ^32^ ^]^ and was ascribed to the phase segregation of the 3D and 2D phases. The film morphology is considerably more uniform and compact with the addition of 3 and 6 mg mL^−1^ MACl additive. Distinct from the recent observation of MACl‐assisted larger grain size for FAPbI_3_‐based 3D perovskites,^[^ ^11^ ^]^ the grain size in the (BDA)(MA)*_n_*_‐1_Pb*_n_* I_3_ *_n_*_+1_ (〈*n*〉 = 4) film decreases with increasing the MACl concentration, and the RMS value is greatly reduced to 14.7 and 11.2 nm. When the MACl concentration further reaches to 10 mg mL^−1^, the film morphology is coarser and the RMS value increases to 18.0 nm, which is ascribed to less compactness and the formation of voids, as evidenced by cross‐sectional SEM analysis (Figure 2f; Figure S3c, Supporting Information). Grains with uniform size are well connected within the optimized film doped with 6 mg mL^−1^ MACl. These observations prove that the prolonged crystallization window is beneficial for suppressing phase segregation of the AA′*_n_*_−1_M*_n_* X_3_ *_n_*_+1_ perovskite.

Figure 2g shows a dominant XRD peak at diffraction angle of 14.1°, assigned to the (111) crystallographic plane for all the films. This indicates that most QWs are aligned orthogonal to the substrate, consistent with the previous reports.^[^ ^20^, ^35^, ^54^ ^]^ Interestingly, the thickness‐corrected intensity of the (111) peak continuously increases with MACl concentration, suggesting an enhanced crystallinity. As can be seen from the full‐width‐at‐half‐maximum (FWHM) of the peak (111) shown in Figure 2h, the FWHM value gradually decreases from 0.284° to 0.194° as MACl increases, suggesting a larger crystallite size. Moreover, there are 2D perovskite (BDA)(MA)*_n_*_‐1_Pb*_n_* I_3_ *_n_*_+1_ (*n* < 4) (0k0) crystal planes, and these peaks disappear with the increase of additive, but the (006) peak strengthened, indicating that the phase purity of perovskite is higher, while growth orientation of QW tends to be more random.^[^ ^62^, ^63^ ^]^ The parallel oriented QWs can inhibit charge transfer along the perpendicular direction. These results further prove that the prolonged crystallization window via additive treatment is beneficial to the growth of QW, but too much additive will make the QW orientation more random. Therefore, the additive concentration must be controlled for the merits of both large crystal size and preferential QW orientation.

### 2.3 Photophysical Properties

**Figure 3:**
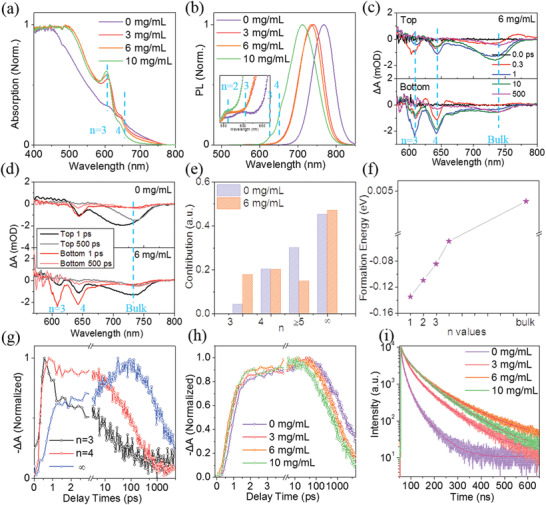
a) UV−vis absorption spectra of the 2D (BDA)(MA)*_n_*_‐1_Pb*_n_* I_3_ *_n_*_+1_ (〈*n*〉 = 4) perovskite films with different additive concentrations. b) Steady‐state photoluminescence (PL) spectra of the 2D (BDA)(MA)*_n_*_‐1_Pb*_n_* I_3_ *_n_*_+1_ (〈*n*〉 = 4) perovskite films with different additive concentrations measured from the top side. c) Transient absorption (TA) spectra at different delay times for optimized (with 6 mg mL^−1^ MACl) film. d) TA spectra at *t* = 1 ps and 500 ps for the control and optimized (with 6 mg mL^−1^ MACl) films under top‐ and bottom‐excitation. e) The composition of QWs in the control and optimized (with 6 mg mL^−1^ MACl) films as estimated from the TA spectroscopy with 1 ps delay time. f) Crystal formation energy of the (BDA)(MA)*_n_*_‐1_Pb*_n_* I_3_ *_n_*_+1_ (*n* = 1−∞) materials from precursors. g) The normalized TA dynamics of the bleaching recovery under bottom‐excitation for the optimized (with 6 mg mL^−1^ MACl) film. h) The TA kinetics of the bulk phase of the series of films. i) Time‐resolved photoluminescence (TRPL) spectra of the films with different additive concentrations.

To gain insight into the alignment of QWs and its dependence on crystallization kinetics, we investigated the distribution of QW thickness and charge transfer of all the films. The UV–vis absorption spectra show that the absorption edge position of the control is close to that of MAPbI_3_ (**Figure** **3**a),^[^ ^7^ ^]^ and there are also increasing excitonic peaks with *n* = 3, 4 at 612 and 649 nm,^[^ ^39^ ^]^ which suggests that the (BDA)(MA)*_n_*_‐1_Pb*_n_* I_3_ *_n_*_+1_ (〈*n*〉 = 4) films are composed of 3D bulk phase and 2D QWs with various thicknesses. We propose that the enhanced low‐*n* excitonic peaks may be caused by the MACl additive changing the thickness of high‐*n* QWs, which will be further discussed later. The photoluminescence (PL) spectra excited from the top shows a blue‐shift similar to the absorption spectra (Figure 3b). The dominant emission peak of high‐*n* QWs gradually shifts from 767 to 741, 735, and 711 nm with the MACl concentration increases, which is consistent with the trend of bandgap (Figure S4, Supporting Information).

The compositional differences for the optimized film treated with 6 mg mL^−1^ MACl are made clear when the transient absorption spectra (TA) under top‐ and bottom‐excitation are compared, as shown in Figure 3c. The bleaching of low‐*n* (*n* = 3, 4) QWs and the bulk phase were observed in all films. It can be confirmed that low‐*n* 2D QWs are more close to the substrate, while high‐*n* QWs are mainly on the film surface. The spectra for other films are shown in Figures S5a–h and S6 (Supporting Information). Such thickness distribution of QWs was also observed previously within (A)_2_A′*_n_*_‐1_M*_n_* X_3_ *_n_*_+1_ perovskite films^[^ ^39^ ^]^ and was ascribed to the solvent–precursor interaction that is key to controlling the rate of QW formation.^[^ ^64^ ^]^ However, a red‐shift of bulk phase bleaching from 747 to 757 nm was noticed after adding MACl, and the bleaching of *n* = 3, 4 QWs is enhanced, but the distribution trend of QW thickness has not changed (Figure 3d). The relative contributions of different QWs were quantified roughly from the TA bottom‐spectra and summarized in Figure 3e.^[^ ^42^, ^44^ ^]^ The high‐*n* (*n* ≥ 5) QWs in the MACl‐treated film are partially converted into low‐*n* QWs and 3D bulk phase, resulting in a finer distribution of QW thickness inside the perovskite film, which further strengthens the proposed view.

Furthermore, we calculated the crystal formation energy (Δ*H*) through the first‐principles density functional theory (DFT). Δ*H* of the (BDA)(MA)*_n_*_‐1_Pb*_n_* I_3_ *_n_*_+1_ (*n* = 1‐*∞*) gradually decreases from 0.004 to ‐0.10, ‐0.17, ‐0.22 and ‐0.27 eV with the *n* value decreases from *∞* (bulk phase) to 4, 3, 2, and 1, respectively (Figure 3f). This suggests that lower‐*n* QWs are more preferable with the extension of nucleation and growth time, which ultimately leads to a sequential crystallization of QWs in the order of *n*. The kinetics‐dependent distribution of QWs thickness was also observed for (A)_2_A′*_n_*_‐1_M*_n_* X_3_ *_n_*_+1_ perovskite films during thermal annealing.^[^ ^65^ ^]^

The TA dynamics of carrier evolution for the optimized film (with 6 mg mL^−1^ MACl) under bottom excitation are shown in Figure 3g, and other cases are shown in Figure S5a′–h′ (Supporting Information). At early times, the rise of TA kinetics reflects the carrier populating at this QW.^[^ ^39^, ^66^, ^67^ ^]^ The rise of high‐*n* QWs and bulk phase kinetics is accompanied by the rapid decay of low‐*n* QWs kinetics, which is in agreement with the observation for (A)_2_A′*_n_*_‐1_M*_n_* X_3_ *_n_*_+1_ perovskites that electrons transfer from low‐*n* to high‐*n* QWs. In addition, the bulk phase kinetics of all films compared in Figure 3h show that the rise rate is faster with the MACl concentration increase. For example, the carrier populating time is ≈14 ps for the optimized film (with 6 mg mL^−1^ MACl), which is much shorter than the control (≈48 ps). This indicates that the finer gradient of QW thickness in the optimized film can effectively promote the charge transfer from low‐*n* QWs to high‐*n* QWs and bulk phase. Furthermore, the energy diagram determined from ultraviolet photoelectron spectroscopy (UPS) shows slight improvements in the highest occupied molecular orbital (HOMO) and the lowest‐unoccupied molecular orbital (LUMO) levels (Figure S7 and Table S5, Supporting Information) for the additive‐treated film, which is expected to improve charge extraction. The average lifetimes determined from the time‐resolved photoluminescence (TRPL) spectroscopy of the bulk phase are 25.0, 71.7, 79.2, and 57.4 ns, respectively (Figure 3i; Table S6, Supporting Information). The result emphasizes the necessity of controlling crystallization kinetics to reduce trap formation and recombination.

### 2.4 Electronic Properties

**Figure 4:**
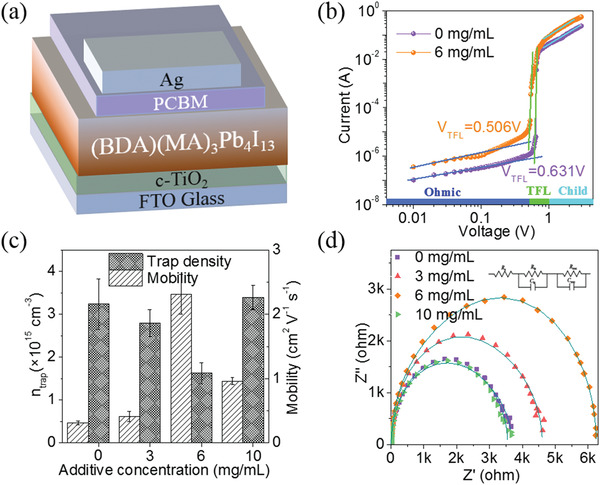
a) Device architecture of a complete solar cell. b) Comparison of the dark *I–V* measurement of the electron‐only devices for the control and optimized (with 6 mg mL^−1^ MACl) films. c) Trap‐state densities and electron mobilities of the films with different MACl concentrations. d) Electrical impedance spectroscopy (EIS) data of the devices with different MACl concentrations, and the inset gives the equivalent circuit for fitting the Nyquist plots.

The trap density and charge mobility were measured for all devices with the architecture of FTO/c‐TiO_2_/perovskite/PCBM/Ag, as shown in **Figure** **4**a. The dark *I−V* characteristics for the control and optimized films (6 mg mL^−1^ MACl) are shown in Figure 4b, and other cases are shown in Figure S8 (Supporting Information). The electron mobilities are 0.3 ± 0.03, 0.4 ± 0.08, 2.3 ± 0.31, and 0.9 ± 0.06 cm^2^ V^–1^ s^–1^ for devices with the MACl concentration increases, respectively, and the corresponding trap densities are 3.2 ± 0.59, 2.8 ± 0.31, 1.6 ± 0.24, and 3.4 ± 0.28 × 10^15^ cm^–3^ (Figure 4c). The higher carrier mobility and lower trap densities for the device (with 6 mg mL^−1^ MACl) indicates more efficient charge transport and suppressed recombination.

We further used electrical impedance spectroscopy (EIS) to characterize the carrier transport properties between the interface of perovskite/charge extraction layers. The series resistance (*R*_s_) values were estimated to be 4.39, 3.34, 3.21, and 7.41 Ω cm^–2^, respectively, and the corresponding recombination resistance (*R*_rec_) are 2881, 3349, 4855, and 2260 Ω cm^–2^ (Figure 4d; Table S7, Supporting Information). Comparing the results, we found that an appropriate amount of additives effectively reduced the *R*_s_ and increased the *R*_rec_ of the device, indicating that MACl can inhibit carrier recombination, enhance the charge transfer and interface contact between perovskite/charge transport layer, and reduce the charge accumulation at the interface, which plays a positive role in improving the FF and *V*_oc_. The device (with 6 mg mL^−1^ MACl) exhibits the lowest *R*_s_ and the highest *R*_rec_, with the highest *V*_oc_ and FF, which are inseparable from the enhancement of perovskite crystallinity and flatness.^[^ ^68^ ^]^

### 2.5 Device Stability

**Figure 5:**
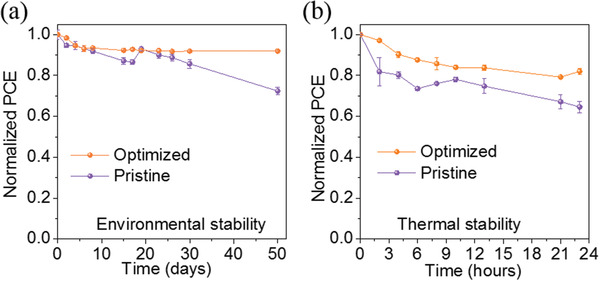
a) Normalized PCEs of the control and optimized (with 6 mg mL^−1^ MACl) devices without encapsulation exposure to ambient conditions (≈30−40% RH, ≈25 °C, dark) for 50 d. b) Normalized PCEs of the corresponding nonencapsulated devices under heating stress at 80 °C for 23 h in an inert atmosphere.

Finally, the environmental stability and thermal stability were tested respectively. **Figure** **5**a records the normalized PCEs of the control and optimized (with 6 mg mL^−1^ MACl) devices without encapsulation for 50 d under ambient conditions (≈30−40% RH, 25 °C, dark). The optimized device (with 6 mg mL^−1^ MACl) retains 92% of its initial PCE, significantly exceeding the control, as evidenced from the XRD analysis (Figure S9, Supporting Information).^[^ ^69^, ^70^ ^]^ As expected, the thermal stability of the optimized (with 6 mg mL^−1^ MACl) device also outperforms the control under the same condition (Figure 5b). The above analysis shows that the MACl are useful in the long‐term environment and thermal stability of the device.

## 3 Conclusion

We have demonstrated important progress in the understanding and control of the formation of QWs and its influence on phase‐alignment and the final optoelectronic properties for the low‐*n* (BDA)(MA)*_n_*_‐1_Pb*_n_* I_3_ *_n_*_+1_ (〈*n*〉 = 4) system. The results show that the MACl additive significantly prolongs the crystallization window during the perovskite deposition. This gives more time for QW nucleation and growth, leading to larger crystal size, less trap formation and finer gradients of the thickness distribution of QWs, which further promote charge transfer and extraction. Finally, the PCE of the additive‐assisted solar cell reached 14.38%, which is one of the high values for the *n* ≤ 4 AA′*_n_*_−1_M*_n_* X_3_ *_n_*_+1_. These important findings on the film formation and phase‐alignment of this type 2D perovskite QWs pave the way towards more efficient and stable optoelectronic devices.

## 4 Experimental Section

### Solution Preparation

The perovskite solution (1.2 m) was composed of BDADI (99.5%, Xi'an Polymer Light Technology Corp.), MAI (99.5%, Xi'an Polymer Light Technology Corp.) and PbI_2_ (99.9985%, Alfa Aesar) (1:3:4 molar ratio) in 1 mL of DMF (99.8%, Aladdin) and 100 µL of DMSO (99.9%, Aladdin) mixed solvents. MACl (99.5%, Xi'an Polymer Light Technology Corp.) was incorporated as an additive into the perovskite precursor with the concentration varying from 0 to 3, 6, and 10 mg mL^−1^. All solutions were filtered prior to solution‐casting. The spiro‐OMeTAD solution was prepared by dissolving 90 mg spiro‐OMeTAD, 22 µL lithium bis(trifluoromethanesulfonyl) imide (99%, Acros Organics, 520 mg mL^−1^) in acetonitrile (99.7+%, Alfa Aesar) and 36 µL 4‐*tert*‐butylpyridine (96%, Aldrich) in 1 mL chlorobenzene. All the solutions were prepared inside a nitrogen glovebox.

### Device Fabrication

The FTO‐coated glass (2.5 cm × 2.5 cm) was cleaned by sequential sonication in acetone, isopropanol, and ethanol for 20 min each and then dried under N_2_ flow and treated by O_3_ plasma for 5 min. The TiO_2_ was prepared by chemical bath deposition with the clean substrate immersed in a TiCl_4_ aqueous solution with the volume ratio of TiCl_4_:H_2_O equal to 0.0225:1 at 70 °C for 1 h. The spin‐coating was accomplished under an inert atmosphere inside a nitrogen glove box. The cleaned FTO/TiO_2_ substrates with TiO_2_ ETLs were exposed to ultraviolet light and ozone for 5 min before spin‐casting. For the hot‐casting process of (BDA)(MA)_3_Pb_4_I_13_ film, the rinsed FTO slides were preheated 100 °C on a hot plate for 10 min immediately before spin‐coating. These hot slides were immediately transferred to the spin‐coating “chuck,” followed by dropping 70 µL precursor solution and spin‐coating at 5000 rpm for 30 s without delay; the color of the thin film turned from pale yellow to brown in few seconds as the solvent escaped. The films were then annealed at 125 °C for 10 min. The hole‐transporting layer was deposited onto the light‐absorbing layer by spin coating spiro‐OMeTAD solution at 5000 rpm for 30 s followed by evaporation of an 80 nm‐thick gold electrode on the top of the cell.

### Optical Metrologies

The in situ absorption test was performed using an F20‐UVX spectrometer (Filmetrics, Inc.) equipped with tungsten halogen and deuterium light sources, with a measurement range of 400–800 nm and a typical sampling time of 0.25. The setup combines an electric motor driving the rotation of a hollow tube capped with a transparent sample holder via a rubber belt connected to the motor. UV–vis absorption spectra were acquired on a PerkinElmer UV‐Lambda 950 instrument. Steady‐state photoluminescence (PL) (excitation at 510 nm, front‐side excitation) and time‐resolved photoluminescence (TRPL) (front‐side) were measured with a PicoQuant FT‐300.

### Transient Absorption Measurements

Femtosecond pump‐probe transient absorption (TA) measurements were performed by using a commercial TA system (Time‐Tech Spectra, LLC). Femtosecond pump‐probe transient absorption (TA) measurements were performed at an appropriate power density (1.81 µJ cm^−2^). The pump pulse with a wavelength of 500 nm and duration of 290 fs generated via a second harmonic generator (SHG) was used to excite all the samples, and the probe beam (from 500 to 850 nm) was detected by a high‐speed spectrometer. The relative presence of each domain was quantified by the amplitude of the transient absorption signal at *t* = 1 ps. All experiments were carried out at room temperature (i.e., *T* = 300 K).

### Electronic Microscopy

The surface morphology and structure were characterized by scanning electronic microscopy (SEM) (FE‐SEM; SU‐8020, Hitachi) and atomic force microscopy (AFM, Dimension ICON), respectively.

### X‐Ray Diffraction (XRD)

XRD measurements were carried out in a *θ*–2*θ* configuration with a scanning interval of 2*θ* between 5° and 50° on a Rigaku Smart Lab (X‐ray source: Cu K*α*; *λ* = 1.54 Å).

### Solar Cell Characterizations

The *J–V* curves of the perovskite solar cells were analyzed using a Keithley 2400 SourceMeter under ambient conditions at room temperature, and the illumination intensity was 100 mw cm^–2^ (AM 1.5G Oriel solar simulator). The scan rate was 0.3 V s^–1^, the delay time was 10 ms, and the scan step was 0.02 V. The power output of the lamp was calibrated against an NREL‐traceable KG5 filtered silicon reference cell. The device area of 0.09 cm^2^ is defined by a metal aperture to avoid light scattering from the metal electrode into the device during the measurement. The EQE was characterized on the QTest Station 2000ADI system (Crowntech. Inc., USA), and the light source is a 300‐W xenon lamp. The monochromatic light intensity for EQE measurement was calibrated using a reference silicon photodiode. All measurements were carried out without any encapsulation in ambient conditions unless otherwise stated.

### Mobility Measurement

Electron‐only devices (FTO/c‐TiO_2_/Perovskite/PCBM/Ag) were fabricated to determine the electron mobility of the devices. The dark *J–V* characteristics of the electron‐only devices were measured on a Keithley 2400 SourceMeter. The mobility is extracted by fitting the *J–V* curves to the Mott‐Gurney equation. The trap‐state density was determined by the trap‐filled limit voltage using the equation in the Supporting Information.

### Electrical impedance Spectroscopy (EIS) Measurement

Electrical impedance spectroscopy (EIS) measurements were conducted using an electrochemical workstation (IM6ex, Zahner, Germany) with the frequency range from 10 Hz to 4 MHz under open‐circuit conditions in the dark.

### Computational Details

The density‐functional theory (DFT) calculations were performed using the Vienna ab initio simulation package (VASP). The projected augmented wave (PAW) method and the Perdew–Burke–Ernzerhof (PBE) functional within the generalized gradient approximation (GGA) were employed to describe the interaction between ion‐cores and valence electrons and the exchange‐correlation effects, and an energy cutoff of 500 eV was set for the plane‐wave function's expansion. The van der Waals (vdWs) dispersion correction was applied and described by the DFT‐D3 correction. A Γ‐centered k‐point sampling of 5 × 5 × 2 for Brillouin zone integration was generated using the Monkhorst‐Pack scheme during the structural optimization of (BDA)(MA)*_n_*_‐1_Pb*_n_* I_3_ *_n_*_+1_ (*n* = 1, 2, 3, 4). The tetragonal phase of MAPbI_3_ is adopted for the bulk calculation with a k‐point sampling of 5 ×5 × 3. The lattice parameters and atomic positions of all the structures were relaxed until the total energy changes were less than 1.0 × 10^–5^ eV and the maximum force component acting on each atom was less than 0.02 eV Å^–1^. The formation energies (Δ*H*) of (BDA)(MA)*_n_*_‐1_Pb*_n_* I_3_ *_n_*_+1_ (*n* = 1, 2, 3, 4) and MAPbI_3_ are calculated according to the following routes:

(1) $$\left(\mathrm{BDA}\right){\left(\mathrm{MA}\right)}_{n-1}Pb_{n}I_{3n+1}\to \mathrm{BDADI}+\left(n-1\right)\mathrm{MAI}+n\mathrm{Pb}I_{2}$$

(2) $$\mathrm{MAPb}I_{3}\to \mathrm{MAI}+\mathrm{Pb}I_{2}$$

## Conflict of Interest

The authors declare no conflict of interest.

## Supporting information

Supporting Information
