# Supplementary material for: Effective Phase‐Alignment for 2D Halide Perovskites Incorporating Symmetric Diammonium Ion for Photovoltaics
Source: Adv Sci (Weinh). 2021 May 24;8(13):2001433. doi: 10.1002/advs.202001433 (PMC8327467; doi:10.1002/advs.202001433)
Supplement: Supplementary file 1 — Supporting Information [file ADVS-8-2001433-s001.pdf]

© 2020 Wiley-VCH GmbH

ADVANCED SCIENCE
Open Access

Supporting Information

for *Adv. Sci.*, DOI: 10.1002/advs.202001433

Effective Phase-Alignment for 2D Halide Perovskites Incorporating Symmetric Diammonium Ion for Photovoltaics

Yalan Zhang,[1] Jialun Wen,[1] Zhuo Xu,[1] Dongle Liu,[1] Tinghuan Yang,[1] Tianqi Niu,[1] Tao Luo,[1] Jing Lu,[1] Junjie Fang,[1] Xiaoming Chang,[1] Shengye Jin,[2] Kui Zhao,[1] and Shengzhong (Frank) Liu,[1,2]**

Supporting Information

Effective Phase-Alignment for 2D Halide Perovskites Incorporating Symmetric Diammonium Ion for Photovoltaics

Yalan Zhang,[1] Jialun Wen,[1] Zhuo Xu,[1] Dongle Liu,[1] Tinghuan Yang,[1] Tianqi Niu,[1] Tao Luo,[1] Jing Lu,[1] Junjie Fang,[1] Xiaoming Chang,[1] Shengye Jin,[2] Kui Zhao,[1] and Shengzhong (Frank) Liu,[1,2]**

Y. Zhang, J. Wen, Z. Xu, D. Liu, T. Yang, T. Niu, T. Luo, J. Lu, J. Fang, X. Chang, Prof. K. Zhao, S. (F.) Liu
[1]Key Laboratory of Applied Surface and Colloid Chemistry, Ministry of Education; Shaanxi Key Laboratory for Advanced Energy Devices; Shaanxi Engineering Lab for Advanced Energy Technology, School of Materials Science and Engineering, Shaanxi Normal University, Xi'an 710119, China.
E-mail: zhaok@snnu.edu.cn
Prof. S. Jin, Prof. S. (F.) Liu
[2]Dalian National Laboratory for Clean Energy; iChEM, Dalian Institute of Chemical Physics, Chinese Academy of Sciences, Dalian, 116023; University of the Chinese Academy of Sciences, Beijing 100039, China.
E-mail: szliu@dicp.ac.cn

Table S1. Summary of the photovoltaic parameters of the devices with different substrate temperatures.

Substrate temperature (°C)	V_{oc} (V)	J_{sc} (mA cm^{-2})	FF (%)	PCE (%)	PCE_{MAX} (%)
80	1.10±0.01	11.9±0.54	50.2±1.02	6.6±0.39	7.13
100	1.10±0.01	15.3±0.28	46.8±1.28	7.9±0.30	8.25
120	1.05±0.01	13.5±0.21	43.0±0.51	6.1±0.12	6.35
150	0.83±0.01	14.3±0.28	42.1±2.08	5.1±0.40	5.38

Table S2. Summary of the photovoltaic parameters of the devices with different annealing temperatures.

Annealing condition (°C)	V_{oc} (V)	J_{sc} (mA cm^{-2})	FF (%)	PCE (%)	PCE_{MAX} (%)
100	1.10±0.01	18.1±0.15	54.8±1.61	10.9±0.42	11.49
125	1.08±0.01	19.2±0.10	61.0±1.17	12.8±0.38	13.40
150	0.98±0.01	17.6±0.23	51.8±2.42	9.0±0.57	10.15

Table S3. Summary of the photovoltaic parameters of the devices with different solvent ratios.

Solvent ratio (F:S)	V_{oc} (V)	J_{sc} (mA cm^{-2})	FF (%)	PCE (%)	PCE_{MAX} (%)
3:7	1.07±0.02	14.6±0.30	45.8±0.83	7.22±0.26	7.81
1:1	1.09±0.01	14.5±0.50	44.0±0.59	6.96±0.24	7.36
7:3	1.10±0.01	15.3±0.51	45.3±0.75	7.69±0.27	8.08
8:2	1.10±0.01	16.5±0.21	51.0±1.24	9.40±0.28	9.98
10:1	1.12±0.01	17.5±0.27	48.5±1.22	9.57±0.26	9.97

Table S4. Summary of the photovoltaic parameters of the devices with different solution concentrations.

Concentration (M)	V_{oc} (V)	J_{sc} (mA cm^{-2})	FF (%)	PCE (%)	PCE_{MAX} (%)
1	1.12±0.01	17.46±0.31	48.1±1.62	9.4±0.37	9.97
1.2	1.11±0.01	17.98±0.32	49.3±1.36	9.8±0.43	10.63

Table S5 Summaries of UPS spectra parameters of the control and optimized (with 6 mg/mL additive) $(BDA)(MA)_{n-1}Pb_nI_{3n+1}$ perovskite films.

Sample	E_{cutoff} (eV)	E_f (eV)	E_v (eV)	E_c (eV)
0 mg/mL	17.146	1.429	5.50	3.88
6 mg/mL	17.136	1.396	5.48	3.80

Table S6. Summaries of fitting parameters for time-resolved photoluminescence (TRPL) for the $(BDA)(MA)_{n-1}Pb_nI_{3n+1}$ perovskite films with different additive concentrations.

Sample	τ_1 (ns)	A1 (%)	τ_2 (ns)	A2 (%)	Average τ (ns)
0 mg/mL	39.7	19.21	8.4	80.79	25.0
3 mg/mL	88.5	43.41	23.7	56.59	71.7
6 mg/mL	97.6	41.34	22.1	58.66	79.2
10 mg/mL	82.2	27.21	20.8	72.79	57.4

Table S7. Summaries of EIS parameters for the devices with different additive concentrations.

Sample	R_s (Ω cm^{-2})	R_{tr} (Ω cm^{-2})	C_{tr} (F cm^{-2})	R_{rec}(Ω cm^{-2})	C_{rec}(F cm^{-2})
0 mg/mL	4.39	792	1.315×10^{-6}	2881	4.148×10^{-8}
3 mg/mL	3.34	1231	3.796×10^{-7}	3349	3.854×10^{-8}
6 mg/mL	3.21	1394	7.327×10^{-8}	4855	5.993×10^{-8}
10 mg/mL	7.41	1280	2.257×10^{-8}	2260	4.485×10^{-8}

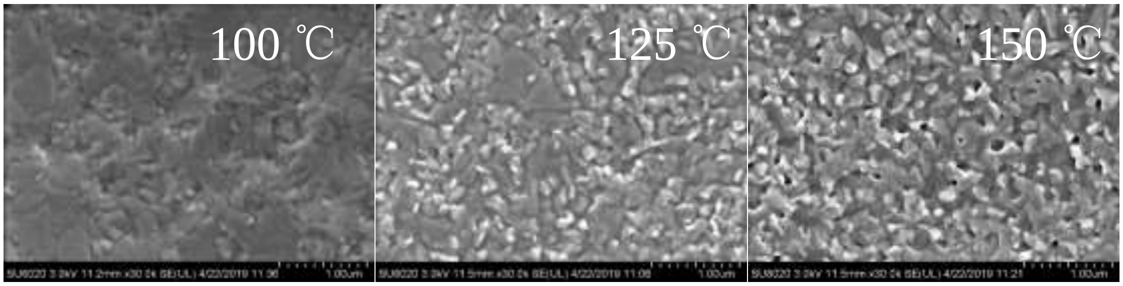

Figure S1. SEM images of thin films (with 6 mg/mL MACl) prepared at different annealing temperatures.

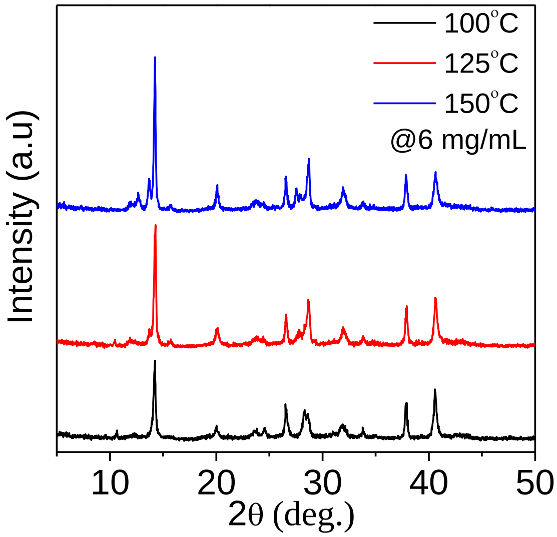

Figure S2. XRD patterns of thin films (with 6 mg/mL MACl) prepared at different annealing temperatures.

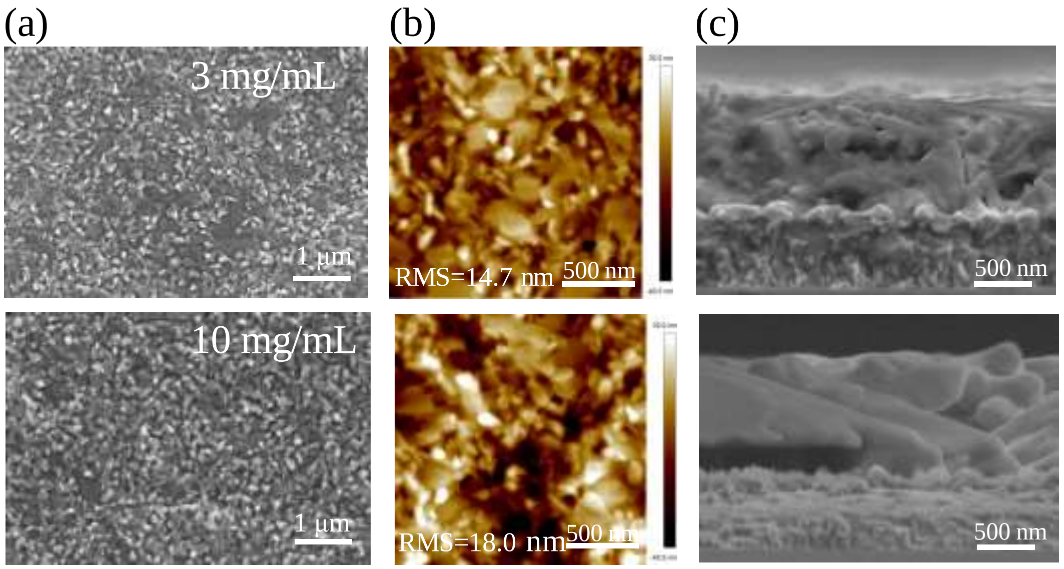

Figure S3. (a) Plan-view scanning electronic microscopy (SEM) images, (b) atomic force microscopy (AFM) images, and (c) cross-sectional SEM images for the films with 3 and 10 mg/mL additive.

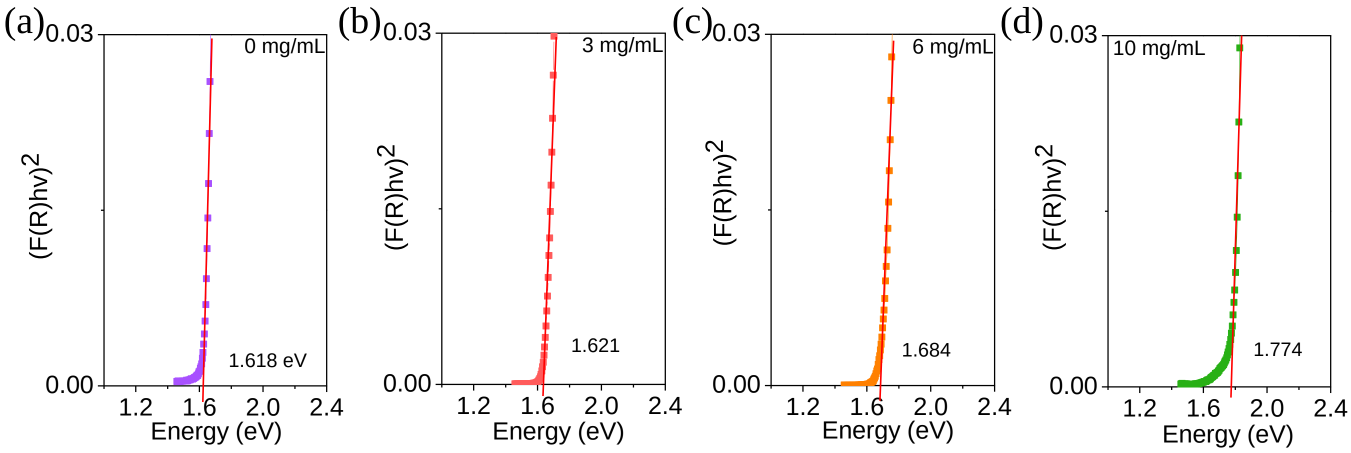

Figure S4. (a-d) Tauc plots showing bandgaps (Eg) for the films with 0, 3, 6, and 10 mg/mL additive, respectively.

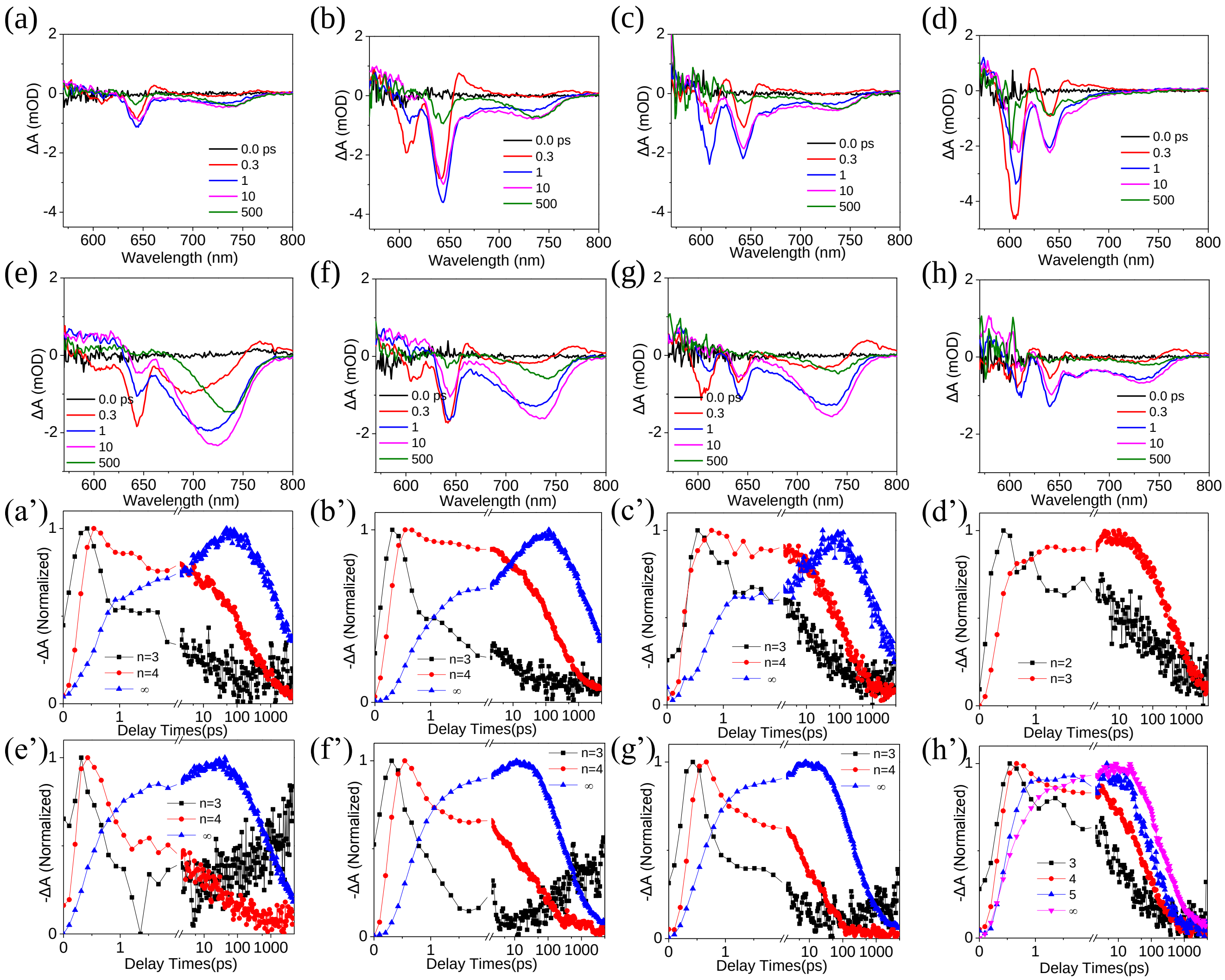

Figure S5. (a-d) Transient absorption (TA) spectra of the films under bottom-photoexcitation with the additive concentrations of 0, 3, 6, and 10 mg/mL, respectively. (e-h) TA spectra of the corresponding perovskite films under top-photoexcitation, respectively. (a’-d’) Normalized TA dynamics for the films under bottom-photoexcitation with the additive concentrations of 0, 3, 6, and 10 mg/mL, respectively. (e’-h’) Normalized TA dynamics of the corresponding perovskite films under top-photoexcitation, respectively.

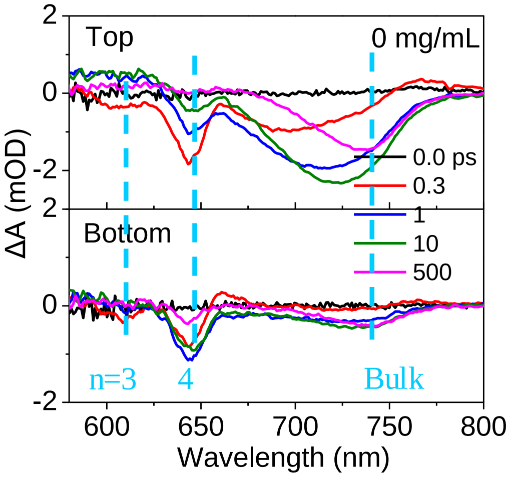

Figure S6. Comparison of transient absorption (TA) spectra at different delay times for the control film.

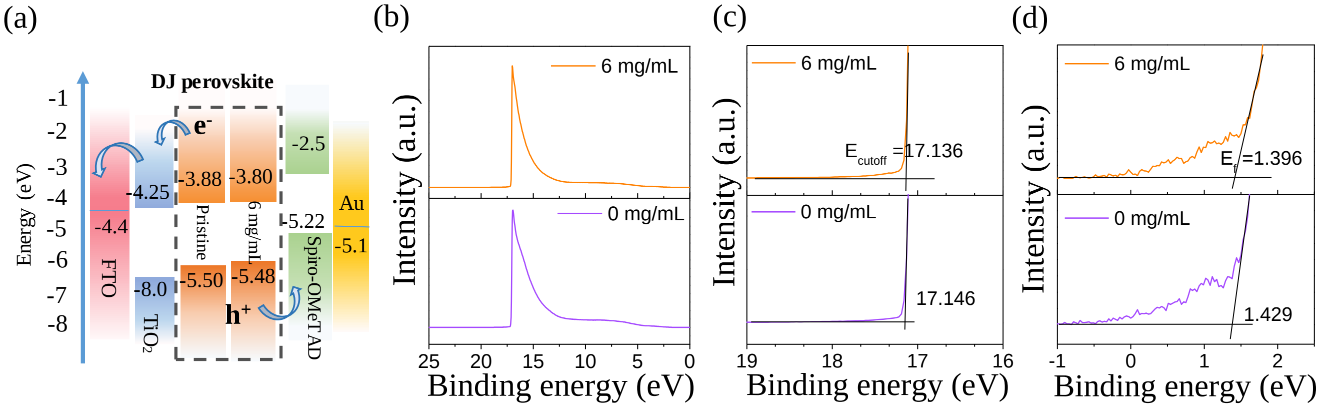

Figure S7. (a) The corresponding energy diagrams in the completed device. The energy levels for the TiO_2 and Spiro-OMeTAD are from a previous report.[1] (b-d) Ultraviolet photoelectron spectroscopy spectra of the control and optimized (with 6 mg/mL MACl) films.

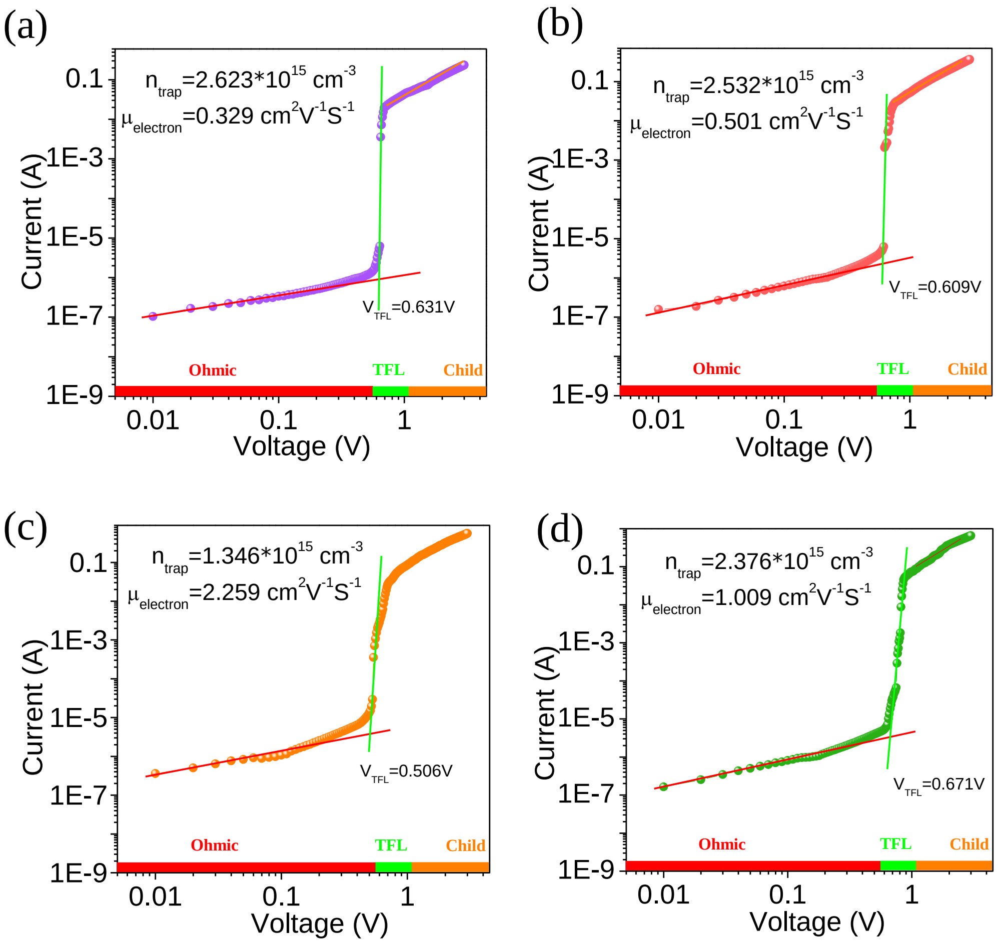

Figure S8. (a-d) Dark *I*–*V* curves of the electron-only devices displaying V_{TFL} kink point behavior for the $(BDA)(MA)_{n-1}Pb_nI_{3n+1}$ perovskite films with additive concentrations of 0, 3, 6, and 10 mg/mL, respectively.

The trap density was determined using the following equation:

$$n_{trap} = \frac{2\varepsilon_0\varepsilon_r V_{TFL}}{eL^2} \quad (1)$$

where ε_0 is the vacuum permittivity, ε_r is the relative dielectric constant, V_{TFL} is the onset voltage of the trap-filled limit region, e is the elementary charge, and L is the distance between the electrodes. The electron mobility was further extracted using the Mott–Gurney Law:[2]

$$\mu = \frac{8J_D L^3}{9\varepsilon_0\varepsilon_r V^2} \quad (2)$$

where J_D is the current density and V is the applied voltage.

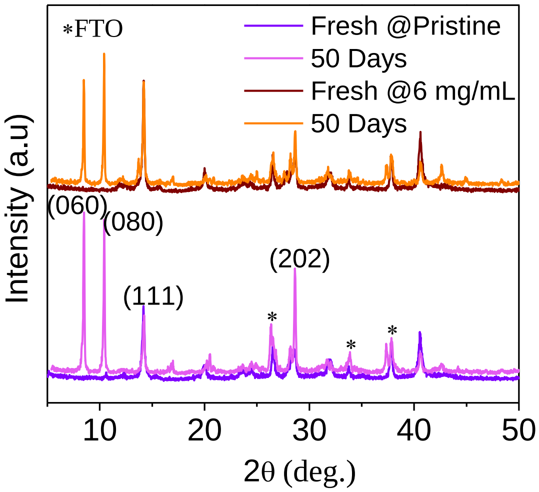

Figure S9. Comparison of XRD patterns of the corresponding perovskite films after 50 days of ambient exposure.

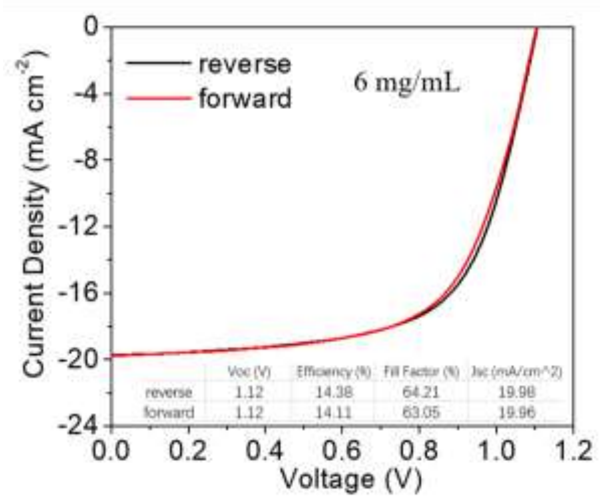

Figure S10 *J*–*V* curves of the representative solar cell measured under different voltage scan directions.

References

(1) Ajay, K. J., Ashish, K., Tsutomu, M., *Chem. Rev*. **2019**, *119*, 3036.

(2) Mott, N. F., Gurney, R. W., Oxford University Press, **1940**.
